# Isobaric Tags for Relative and Absolute Quantitation (iTRAQ)-Based Comparative Proteome Analysis of the Response of Ramie under Drought Stress

**DOI:** 10.3390/ijms17101607

**Published:** 2016-09-27

**Authors:** Xia An, Jingyu Zhang, Lunjin Dai, Gang Deng, Yiwen Liao, Lijun Liu, Bo Wang, Dingxiang Peng

**Affiliations:** 1 Key Laboratory of Crop Ecophysiology and Farming Systems in the Middle Reaches of the Yangtze River, Ministry of Agriculture, College of Plant Science and Technology, Huazhong Agricultural University, Wuhan 430070, China; anxia@webmail.hzau.edu.cn (X.A.); zhangjingyu0408@gmail.com (J.Z.); dailunjin@gmail.com (L.D.); lywen0902@gmail.com (Y.L.); liulijun@mail.hzau.edu.cn (L.L.); 2 Flower Research and Development Centre, Zhejiang Academy of Agricultural Sciences, Hangzhou 311202, China; 3 School of Agricultural Science, Yunnan University, Kunming 650091, China; denggang1986@ynu.edu.cn

**Keywords:** comparative proteome analysis, drought stress, ramie, isobaric tags for relative and absolute quantitation (iTRAQ)

## Abstract

In this study, we conducted the first isobaric tags for relative and absolute quantitation (*isobaric tags for relative and absolute quantitation* (iTRAQ))-based comparative proteomic analysis of ramie plantlets after 0 (minor drought stress), 24 (moderate drought stress), and 72 h (severe drought stress) of treatment with 15% (*w*/*v*) poly (ethylene glycol)6000 (PEG6000) to simulate drought stress. In our study, the association analysis of proteins and transcript expression revealed 1244 and 968 associated proteins identified in leaves and roots, respectively. L1, L2, and L3 are leaf samples which were harvested at 0, 24, and 72 h after being treated with 15% PEG6000, respectively. Among those treatment groups, a total of 118, 216, and 433 unique proteins were identified as differentially expressed during L1 vs. L2, L2 vs. L3, and L1 vs. L3, respectively. R1, R2, and R3 are root samples which were harvested at 0, 24, and 72 h after being treated with 15% PEG6000, respectively. Among those treatment groups，a total of 124, 27, and 240 unique proteins were identified as differentially expressed during R1 vs. R2, R2 vs. R3, and R1 vs. R3, respectively. Bioinformatics analysis indicated that glycolysis/gluconeogenesis was significantly upregulated in roots in response to drought stress. This enhancement may result in more glycolytically generated adenosine triphosphate (ATP) in roots to adapt to adverse environmental conditions. To obtain complementary information related to iTRAQ data, the mRNA levels of 12 proteins related to glycolysis/gluconeogenesis in leaves and 7 in roots were further analyzed by qPCR. Most of their expression levels were higher in R3 than R1 and R2, suggesting that these compounds may promote drought tolerance by modulating the production of available energy.

## 1. Introduction

Among several factors controlling plant growth, water plays a vital role [1]. A global water shortage is a very serious environmental problem. A looming water crisis, which is lead by poor water management, increased competition of limited water resources, and the uncertain consequences of global warming, is threatening agricultural productivity world widely [2]. With increasingly limited water resources of agriculture, developing the tolerance of crops to water shortages might be the most economical way to improve agricultural productivity [3]. Therefore, an urgent need exists to enhance crop tolerance to drought stress.

Plants can respond to adverse environments with several physiological and biochemical strategies that were derived from a long-term domestication process [2]. Plant response to drought stress is a complex course, and several mechanisms include drought resistance, which include drought escape via a developmental plasticity, drought avoidance through reducing water loss and enhancing water uptake, and drought tolerance by means of antioxidant capacity and osmotic adjustment [4]. In order to defend against drought stress, plants undergo a process of stress acclimation. This process may require changes in a large number of stress-related gene expressions [5,6,7] and synthesis of diverse functional proteins [8,9,10]. Recently, expanding transcriptome data sets has uncovered that many genes were induced or repressed upon drought stress in *Arabidopsis* [11], maize [12], rice [13], soybean [14], and ramie [15]. This study was lead to an understanding of the drought stress regulatory mechanism. However, several transcripts will experience transcriptional, translational, and post-translational modifications, revealing that the potential drought stress molecular mechanisms via differentially expressed gene identification are not comprehensive enough [16].

Ramie (*Boehmeria nivea* L.) is an important natural fiber crop. Ramie is grown on about 80,000 ha with an annual fiber production of 150,000 t in 2012 (FAOSTAT, http://faostat3.fao.org). In China, ramie is the second most important fiber crop, behind cotton in crop acreage and fiber production [15]. Ramie grows vigorously in well-watered cultivation environments, resulting in a high yield of vegetative fiber extracted from stem bast. Good irrigation is essential for this crop, as fiber yield is reduced under drought stress [17]. To avoid competing land with food crops, ramie can be transferred to arid or semiarid hilly mountainous areas, where it will face a more serious drought threat in the future. High fiber yields were obtained in drought-tolerant cultivars of ramie with root systems, leaf responses, cellular responses, and biochemical activities that allowed high levels of photosynthesis and carbon deposition under stress [17]. Twelve transcription factors involved in the drought response were found by Illumina tag-sequencing and qRT-PCR in ramie [15]. However, the levels of mRNA and proteins did not always correlate well [10]. Protein expression changes in response to drought stress have been studied in some other plants, and drought stress-induced proteins involved in photosynthesis [18], signaling pathways [19], oxidative stress detoxification [20], and transport [21] have been identified. However, the specific proteins induced in ramie under drought conditions remain unknown.

Here, we provide an iTRAQ-based comparative analysis of the drought-resistant response of ramie. This is the first use of iTRAQ to research on the molecular mechanisms of ramie related to drought stress. Identifying and quantifying multiple sample proteins simultaneously is the advantage of this approach [22]. Proteins that are too large or small, too acidic or basic, too hydrophobic, or in low abundance are difficult to observe via 2D gel electrophoresis, but can be identified by iTRAQ [22,23]. We imposed drought stress by PEG to evaluate plant drought-tolerance preliminarily [24]. In this study, the leaves and roots of “Huazhu No. 5” were harvested 0 (L1 and R1), 24 (L2 and R2), and 72 (L3 and R3) hours after being treated with 15% (*w*/*v*) PEG6000. Our results provide new insights into the ramie response to drought stress.

## 2. Results and Discussion

### 2.1. Analytical Strategy for Proteome Identification under Drought Stress

**Figure 1:**
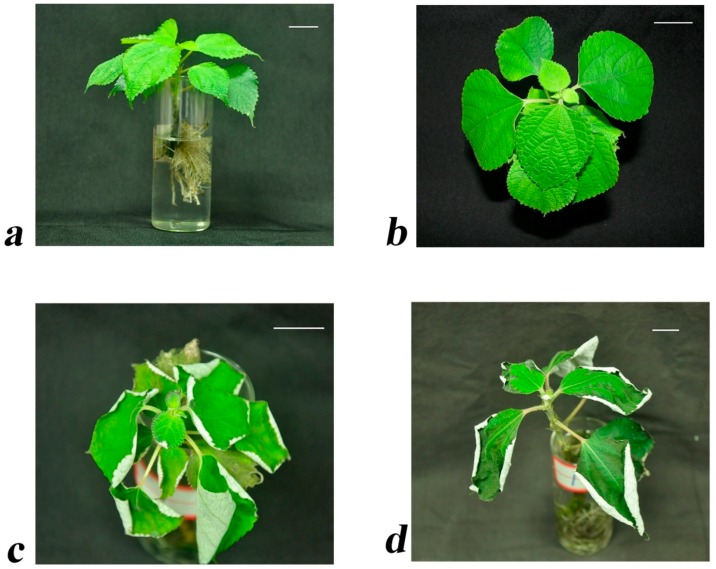
(**a**) After two-week propagation from stem cuttings, the plantlets of “Huazhu No. 5” were transplanted into half-strength Hoagland's solution for 20 days. Leaf and root samples of the same sizes were harvested at 0 h (**b**), 24 h(**c**), and 72 h (**d**) after materials had been treated with 15% (*w*/*v*) PEG6000 to induce drought stress. They were cultured under cool white fluorescent light in 16/8 h (light/dark) with a relative humidity of 50%–70% and temperatures about 25 ± 2 °C in the daytime and 20 ± 2 °C at night. Scale bar = 2 cm.

The leaves only slightly yellowed and curled after 24 h of drought treatment (Figure 1c) but showed severe chlorosis and stopped growing after 72 h (Figure 1d). The relative water content (RWC) of leaves seemed to decline consistently [25].

Many abiotic stresses trigger the production of reactive oxygen species (ROS), which disrupts normal metabolism by causing oxidative damage to membrane lipids, proteins, and nucleic acids [26]. Peroxidase activity (POD) can eliminate these harmful molecules [27]. The POD activity first increased but later decreased [25]. Based on the critical time-points for RWC and the POD activity [25], three critical time-points (0, 24, and 72 h after drought stress) were screened for morphological (Figure 1) and physiological results [25]. An iTRAQ-based quantitative proteome analysis was performed for a global view of the proteome responses to different durations of drought treatments (Figure S1). Protein mass distribution in ramie is shown in Figure S2.

### 2.2. Correlation Coefficients of Biological Replicates

**Figure 2:**
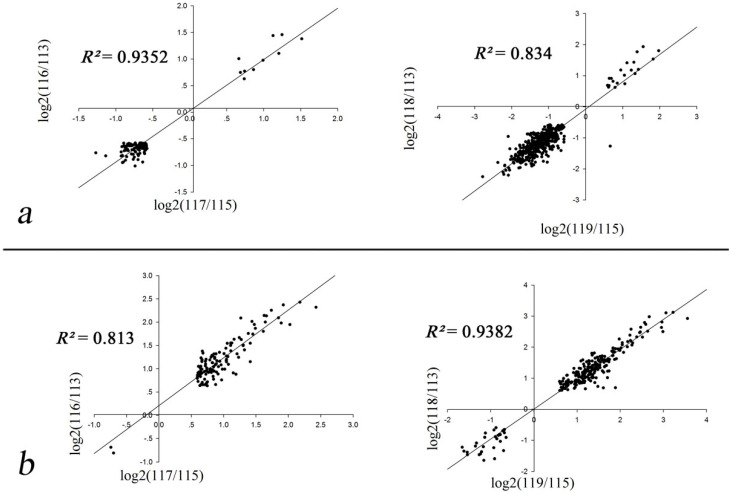
The correlation coefficients were calculated between two biological replicates. The ratios of quantified proteins were log-transformed and plotted. (**a**) Leaves; (**b**) roots. Tags 113 and 115 in Figure 2a represent repeat (1) and repeat (2) of 0 h of leaf treatment with 15% (*w*/*v*) PEG6000, respectively. Tags 116 and 117 in Figure 2a represent repeat (1) and repeat (2) of 24 h of leaf treatment with 15% (*w*/*v*) PEG6000, respectively. Tags 118 and 119 in Figure 2a represent repeat (1) and repeat (2) of 72 h of leaf treatment with 15% (*w*/*v*) PEG6000, respectively. Tags 113 and 115 in Figure 2b represent repeat (1) and repeat (2) of 0 h of root treatment with 15% (*w*/*v*) PEG6000, respectively. Tags 116 and 117 in Figure 2b represent repeat (1) and repeat (2) of 24 h of root treatment with 15% (*w*/*v*) PEG6000, respectively. Tags 118 and 119 in Figure 2b represent repeat (1) and repeat (2) of 72 h of root treatment with 15% (*w*/*v*) PEG6000, respectively. The ratios (L2L3 vs. L1 and R2R3 vs. R1) were then log-transformed and plotted against each other.

To determine differentially expressed proteins in leaves (L1, L2, and L3) and roots (R1, R2, and R3), the correlation coefficients between pairs of biological replicates were first evaluated (Figure 2). The two L1 and R1 controls were used as denominators, respectively. We used the proteins that were quantified with iTRAQ ratios to calculate correlation coefficients. The ratios (L2L3 vs. L1 and R2R3 vs. R1) were then log-transformed and plotted against each other. As illustrated in Figure 2, all correlation coefficients of the biological replicates were equal to or greater than 0.8 [28], indicating the excellent biological reproducibility of drought-regulated protein expression.

### 2.3. Functional Classification and Annotation 

**Figure 3:**
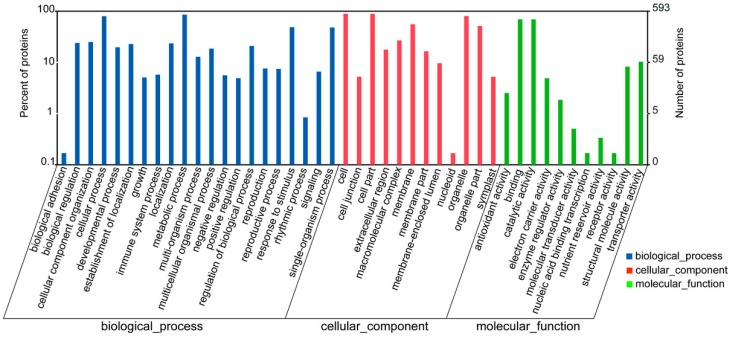
The categorization of proteins is based on gene ontology (GO) annotation. The category number is displayed with biological process, cellular components, and molecular functions. *y*-axis (**left**) represents percentages of proteins identified, *y*-axis (**right**) represents the protein number.

We conducted gene ontology (GO) functional annotation analysis for all identified proteins. The results cover a wide range of biological processes, cellular components, and molecular functions, including 44 important functional groups (Figure 3; Table S1). The largest subcategory in the biological process category was “metabolic processes” and the second was “cellular processes”. In the cellular component category, “cell”, “cell part”, and “organelle” were the main categories. “Binding” and “catalytic activity” were the main categories of molecular function.

**Figure 4:**
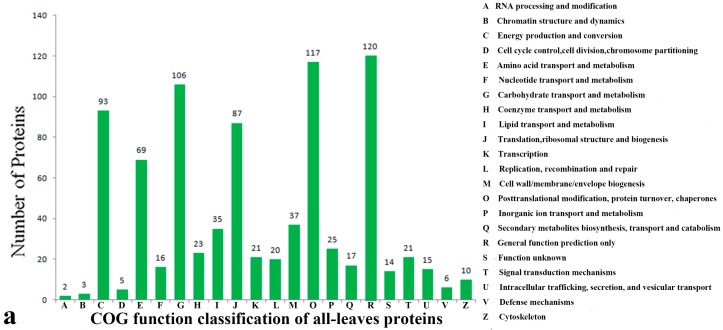

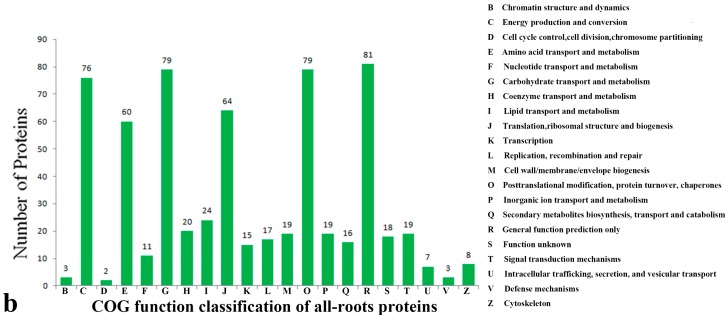
COG function classification of all leaf (**a**) and root (**b**) proteins.

Identified proteins were classified according to their biological functions using the NCBI COG (Cluster of Orthologous Groups of proteins) database (http://www.ncbi.nlm.nih.gov/COG/). All leaf proteins were classified into 22 COG subcategories (Figure 4a; Table S2) including general function prediction only (13.92%); posttranslational modification, protein turnover, and chaperones (13.57%); carbohydrate transport and metabolism (12.30%); energy production and conversion (10.79%); and translation, ribosomal structure, and biogenesis (10.10%). All root proteins were classified into 21 subcategories (Figure 4b; Table S2) including general function prediction only (12.66%); carbohydrate transport and metabolism (12.34%); posttranslational modification, protein turnover, and chaperones (12.34%); energy production and conversion (11.87%); translation, ribosomal structure, and biogenesis (10.00%), and amino acid transport and metabolism (9.38%). In this study, the identified proteins were mainly involved in general function prediction only (R); carbohydrate transport and metabolism (G); energy production and conversion (C), posttranslational modification, protein turnover, and chaperones (O); translation, ribosomal structure, and biogenesis (J), and amino acid transport and metabolism (E) (Figure 4). Proteins related to energy metabolism, stress resistance, “cell growth, differentiation and structure”, and metabolism-related proteins have also been reported in ramie under N, P, and K deficiency [29]. The other identified proteins with COG categories are shown in Figure 4.

### 2.4. Effects of Drought Stress on Expression Changes of the Ramie Leaf and Root Proteomes

The results showed that most proteins in leaves were downregulated under drought stress. Compared to L1 (control), 10 proteins in L2 were upregulated, and 108 proteins were downregulated, while 20 proteins in L3 were upregulated, and 413 were downregulated (Table 1). The upregulated proteins in L3 included all 10 that were upregulated in L2, and the downregulated proteins in L3 included 107 that were also downregulated in L2. Proteins that were upregulated or downregulated in L3 were selected for further analysis.

In contrast, most root proteins were upregulated under drought stress. Compared to R1 (control), 122 proteins in R2 were upregulated, and 2 were downregulated; 211 proteins in R3 were upregulated, and 29 were downregulated (Table 1). The upregulated proteins in R3 included all 122 proteins that were upregulated in R2, and the downregulated proteins in R3 contained 2 proteins that were downregulated in R2. Proteins that were upregulated or downregulated in R3 were selected for further analysis.

**Figure 5:**
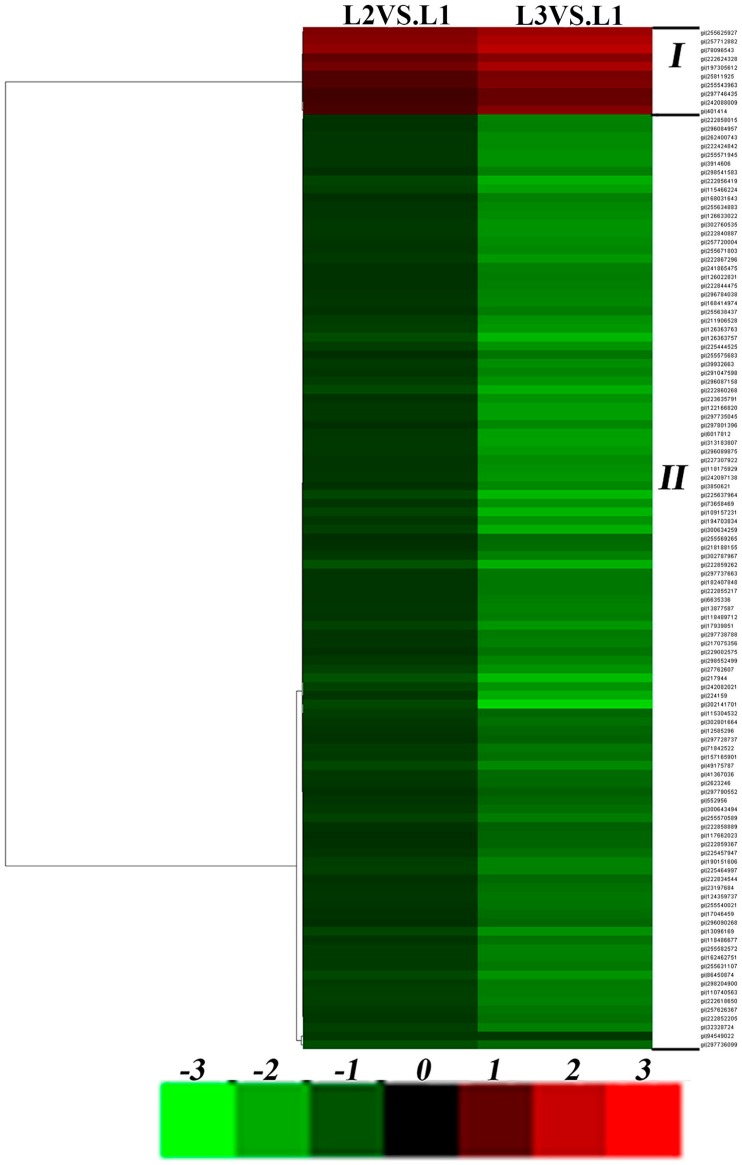
Hierarchical display of data from differentially expressed protein of leaves under drought stress. Upregulated proteins are in red; downregulated proteins are in green (for interpretation of the color references in the figure legend).

**Figure 6:**
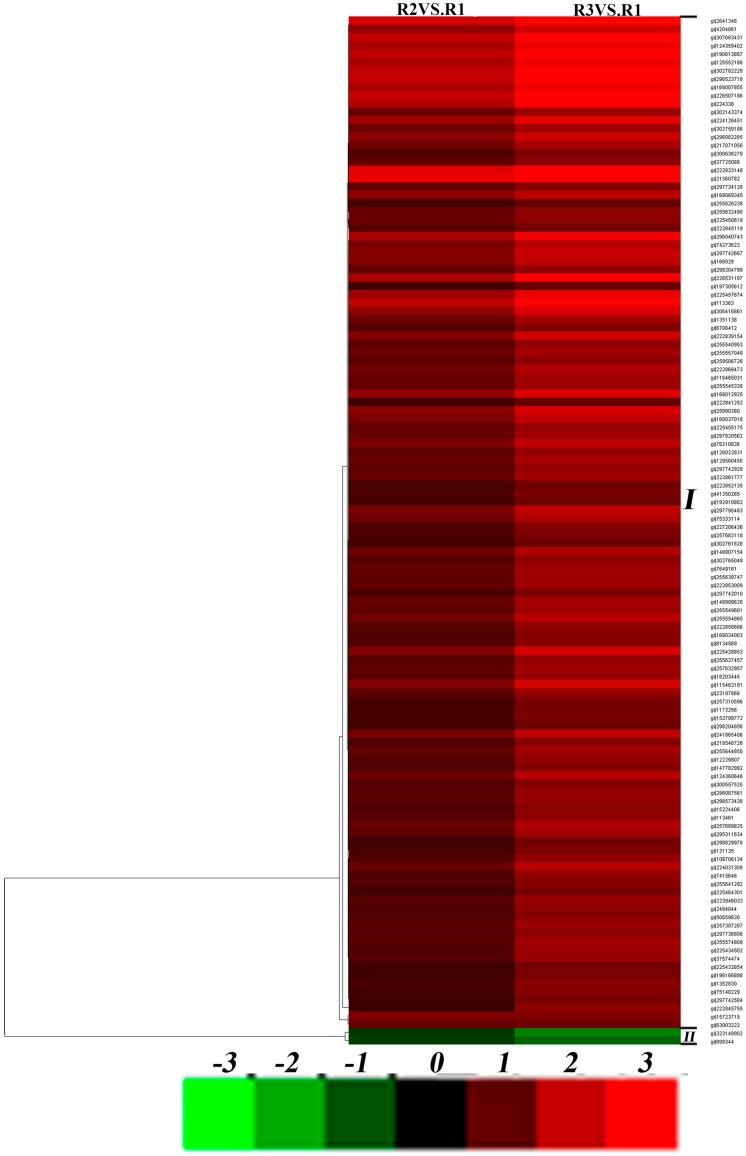
Hierarchical display of data from differentially expressed protein of roots under drought stresses. Upregulated proteins are in red; downregulated proteins are in green (for interpretation of the color references in the figure legend).

The differentially regulated proteins from leaves and roots were clustered according to similarities in change profiles across all conditions. A dendrogram and colored image were produced as a cluster analysis of different samples using Cluster 3.0 (Michael Eisen, Stanford, CA, USA). Dark boxes indicate no change in expression pattern compared to the control. In Figure 5 (Table S3) and Figure 6 (Table S4), each row represents a single protein and each column represents a treatment (Figure 5: L2 on left, L3 on right; Figure 6: R2 on left, R3 on right). Cluster analysis revealed that the differentially expressed proteins in leaves could be generally divided into two groups: continuously upregulated (4.6%; Cluster I) and downregulated (95.4%; Cluster II) in response to drought stress (Figure 5). The differentially expressed proteins in roots also formed two upregulated (87.9%; Cluster I) and downregulated (12.1%; Cluster II) groups in response to drought stress (Figure 6). For Cluster II, proteins in ramie leaves were mainly involved in energy metabolism and photosynthesis. Other protein functions included secondary metabolism, starch and sucrose metabolism, disease/defense, signal transduction, cell structure, and protein synthesis. Plants need a considerable amount of ATP for sufficient energy for growth, development, and stress responses [30]. Under drought stress, the ATP synthesis process in ramie was influenced significantly. Large quantities of ATP-related proteins were downregulated, including ATP synthase alpha subunit, ATP synthase beta subunit, and ATP synthase CF1 alpha subunit. When plants are under abiotic stress, the initial response is to lower energy metabolism by reducing ATP synthesis in cells. In accordance with these results, protein abundance of ATP synthase was decreased under drought stress in spring wheat varieties Ningchun 4 [31]. Photosynthesis is sensitive to drought and other types of stress (e.g., nutrient stress) [29]. For Cluster I, proteins in ramie roots were mainly involved in energy metabolism. Other protein functions included secondary metabolism, amino acid metabolism, disease/defense, sucrose metabolism, and protein synthesis. Large quantities of ATP-related proteins were upregulated, including ATP synthase alpha subunit, ATP synthase beta subunit, and V-type proton ATPase catalytic subunit A. ATP synthase beta subunit was upregulated in drought-tolerant Tibetan wild barley genotype XZ5 but downregulated in drought-sensitive XZ54 [32]. In addition, roots and leaves of ramie also showed differential responses in accumulation of ATP synthesis-related proteins. It has been reported that these enzymes play an important role in the removal of abnormal or damaged proteins and in the fine control of some key cellular components, combining a peptidase and a chaperone activity [33]. Energy deprivation is a general symptom of photosynthetic plants under stress and ultimately arrests growth and causes cell death. Many proteins were upregulated in roots under drought stress to induce alternative glycolysis pathways to maintain energy levels. Energy deficit often enhances inherent pathways of carbohydrate metabolisms [34].

### 2.5. Association and Differential Expression Analysis of Proteome and Transcriptome Data

**Figure 7:**
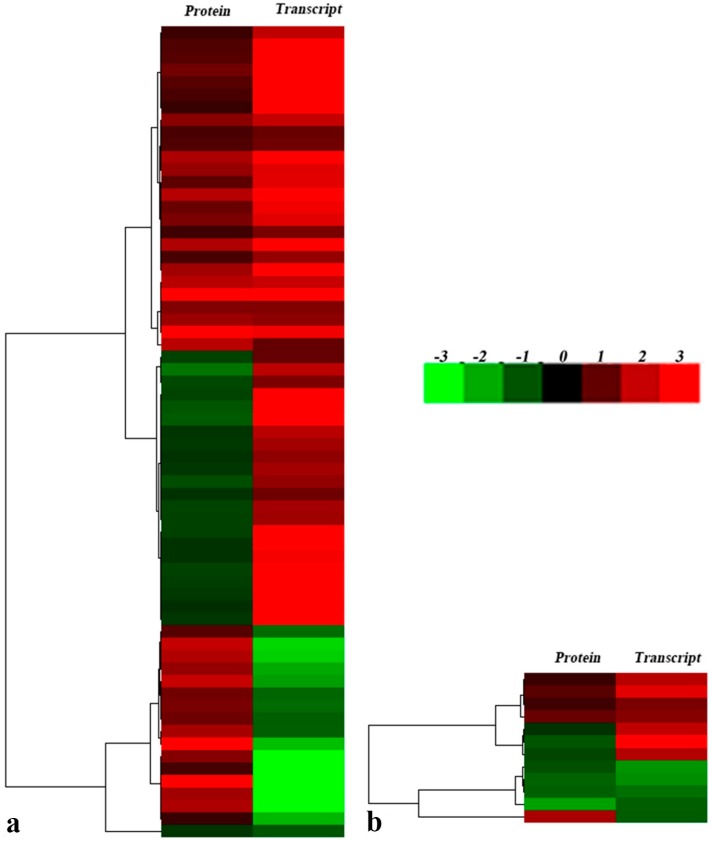
Association clustering analysis of differentially expressed proteins and its corresponding transcripts. Protein (**left**) represents the expression levels of differentially expressed proteins, Transcript (**right**) represents the expression profile of the corresponding genes encoding differentially expressed proteins. (**a**) leaf of ramie; (**b**) root of ramie. Upregulated proteins are in red; downregulated proteins are in green (for interpretation of the color references in the figure legend).

To investigate association and differential expression analysis of proteome data produced in this research and transcriptome data produced in our earlier published Illumina Paired-End sequencing project, hierarchical cluster analyses were conducted in this study. All differently expressed proteins were represented including data from L3 and R3. Approximately, 138,000 transcripts were detected from ramie under PEG6000 simulated drought stress in our earlier published Illumina Paired-End sequencing project. The expression profiles of all differentially expressed proteins and corresponding transcripts under drought stress are shown in Figure 7. The correlations of gene expression at the transcript and protein levels for the leaves and roots were 65 and 12, respectively (Table S5). The results indicated that more differentially expressed proteins were observed in ramie leaves than in ramie roots. Differentially expressed proteins in ramie leaves were mainly involved in photosynthesis and energy metabolism, disease/defense, cell structure, and protein synthesis. It is well known that the photosynthetic system and its maintenance will badly affect plant survival under abiotic stress environment [35,36]. Among the differentially expressed proteins, it was found that photosystem II (PSII) proteins were downregulated in transcription and protein levels. Drought stress has been reported to damage the photosynthetic system by causing severe disruption of the PSII complex [37] and the chloroplast envelope [38]. Similar studies have been reported in rice under drought stress [37]. Water, as the reducing agent, involves absorbed photons to provide fundamental energy for photosynthesis in green plants, so drought might be the most intense of all abiotic stresses affecting the photosynthesis process. In the transcriptional level, ATP synthase CF1 α subunit and ATP synthase γ chain chloroplastic-like isoform 1 were found to be upregulated, but downregulated in the protein level. Fructose 1,6-bisphosphate aldolase was found to be upregulated at the transcriptional level but at the protein level be downregulated. We found that most proteins differentially expressed under drought stress showed contrary trends with their corresponding transcripts (Figure 7) in ramie leaves. Plants may respond to drought stress by changing post-transcriptional regulation. Post-transcriptional regulation is a potential target mechanism that can be deeply studied in order to elucidate the drought response in plants [39]. We found that most proteins differentially expressed under drought stress showed similar trends with their corresponding transcripts (Figure 7) in ramie roots. Chitinase was found to be upregulated at the transcriptional and protein levels in ramie roots. Previous research has indicated that the expression level of the drought-induced protein 3 (DIP3) protein obtained in roots of upland rice was up in a short time under drought tolerance. Thus, our results reveal that the class III chitinases member DIP3 may be a stress-induced protein when plants respond to stress conditions [40].

### 2.6. Identification Biochemical Reactions Significantly Upregulated in Roots by Drought Stress

ABA plays an important role in the expression of genes related to drought stress in nearly all cells [41,42,43]. Drought-stressed ramie roots upregulated heat shock protein (gi|4204861 and gi|255582806), ribosomal protein (gi|241865406, gi|18203445, gi|148807154, gi|50659630, and gi|133793), ubiquitin-conjugating enzyme family protein (gi|192910862), and chaperonin (gi|108706134), all of which involved ABA signaling to enhance the cellular dehydration tolerance. In addition, many ribosome proteins have been linked to cell structure, protein translation, protein biosynthesis, and plant development in wheat [44]. Heat shock proteins play broad roles in many cellular processes in *Arabidopsis* subjected to heat stress [45].

Excessive accumulation of ROS under drought stress can disrupt normal metabolism by oxidative damage of membrane lipids, proteins, and nucleicacids [25]. Catalase (CAT) and POD were all upregulated at the protein level under drought stress. CAT and POD (among other so-called scavengers) are able to eliminate these harmful molecules. Therefore, the mechanisms of the ROS-reducing system and the antioxidant enzyme-increasing system can play important roles in enhancing tolerance to drought stress.

**Figure 8:**
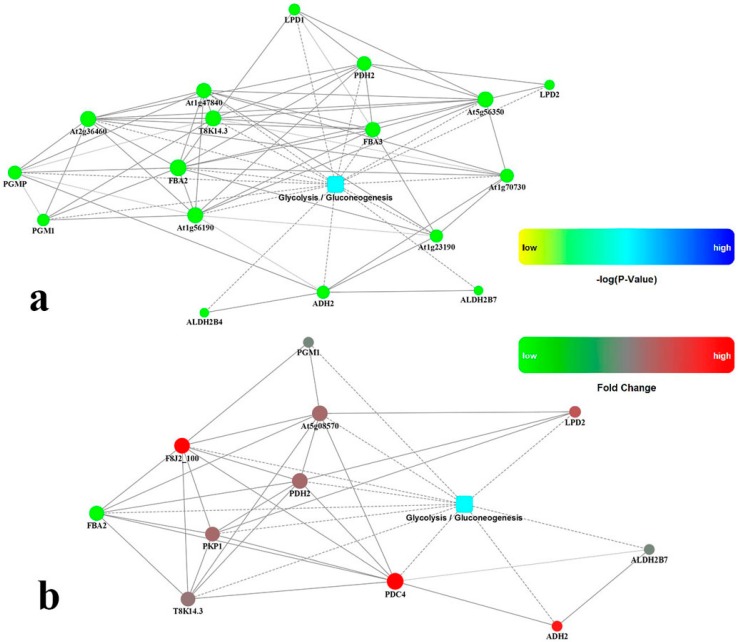
Regulatory changes in the pathway of glycolysis/gluconeogenesis. Colored circle nodes correspond with the ramie genes detected in the isobaric tags for relative and absolute quantitation (iTRAQ) data. (**a**) upregulated or downregulated genes in leaves; (**b**) upregulated or downregulated genes in roots. The above network model is generated with a cytoscape web application, based on information gained from up to four levels of functional analysis: fold change of gene/protein, protein-protein interaction, Kyoto Encyclopedia of Genes and Genomes (KEGG) pathway enrichment, and biological process enrichment. Circle nodes: genes/proteins; rectangle nodes: KEGG pathway or biological process. Pathways are colored in a gradient color from yellow to blue; yellow indicates a lower *p*-value, and blue indicates a higher *p*-value. Biological processes are colored in red. In the case of fold change analysis, genes/proteins are colored in red (upregulation) and green (downregulation). A default confidence cutoff of 400 was used: interactions with a higher confident score are shown as solid lines between genes/proteins; dashed lines indicate otherwise.

Kyoto Encyclopedia of Genes and Genomes (KEGG) Orthology-Based Annotation System (http://kobas.cbi.pku.edu.cn) [46], was used to identify significant pathways involved in the response to drought stress in ramie. The drought-responsive proteins in roots represented a wide range of pathways, including metabolic pathways, glycolysis/gluconeogenesis, biosynthesis of secondary metabolites, ribosomes, oxidative phosphorylation, pyruvate metabolism, glyoxylate and dicarboxylate metabolism, phenylalanine metabolism, phenylpropanoid biosynthesis, and the citrate cycle (TCA cycle). Energy deprivation is a general symptom of stressed photosynthetic plants [47]. Photosynthesis, respiration rates, or both are dramatically reduced under stress [47,48,49], causing energy deprivation and growth arrest [34,49]. The energy deprivation often enhances inherent pathways of carbohydrate metabolism and induces alternative pathways of glycolysis to maintain energy [34]. Glycolysis/gluconeogenesis is an alternative bioenergetic pathway in stressed organisms (Table S6). To obtain information complementary to the iTRAQ data, the mRNA levels of 12 proteins in leaves and 7 proteins in roots related to glycolysis/gluconeogenesis were further analyzed by qPCR. Details of the glycolytic/gluconeogenetic pathway are shown in Figure 8. Eleven proteins in leaves, gi|168035690 (EC:5.4.2.2), gi|302142655 (EC:2.7.1.1), gi|297735045 (EC:4.1.2.13), gi|302774424 (EC:4.1.2.13), gi|82941449 (EC:4.1.2.13), gi|298541583 (EC:2.7.2.3), gi|129915 (EC:2.7.2.3), gi|222868326 (EC:5.4.2.1), gi|297746511 (EC:2.7.1.40), gi|118489203 (EC:1.8.1.4), and gi|298552499 (EC:1.2.1.3), were involved in glycolysis/gluconeogenesis (green in Figure 8). Six proteins in roots, gi|118481158 (EC:2.7.2.3), gi|296523718 (EC:4.1.1.1), gi|298552499 (EC:1.2.1.3), gi|2641346 (EC:1.1.1.1), gi|222845119 (EC:1.2.4.1), and gi|225450619 (EC:1.8.1.4), were also involved (red in Figure 8), while one protein, gi|297735045 (EC:4.1.2.13) in roots, was upregulated and colored by green. The upregulated expressions of these proteins may play a vital role in initiating the glycolytic/gluconeogenetic pathway under unfavorable conditions. This hypothesis is consistent with a previous observation that several glycolysis/gluconeogenesis-related genes were induced under aluminum stress in wheat [50]. Glycolysis could also be used to generate ATP to meet the energy requirement [51]. Under drought treatments, 30% of ramie proteins were related to metabolism and energy conversion. Roots of ramie appear to be able to perceive and convert stress signaling into energy status and induce alternative metabolic pathways to adjust their growth and development in response to drought stress [34]. Thus, enhancement of the glycolytic/gluconeogenetic pathway could result in more glycolytically generated ATP in roots to adapt to adverse environmental conditions [52].

### 2.7. Verification of Isobaric Tags for Relative and Absolute Quantitation (iTRAQ) Data on Selected Candidates by qPCR

**Figure 9:**
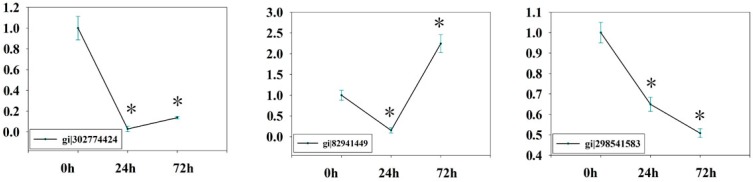

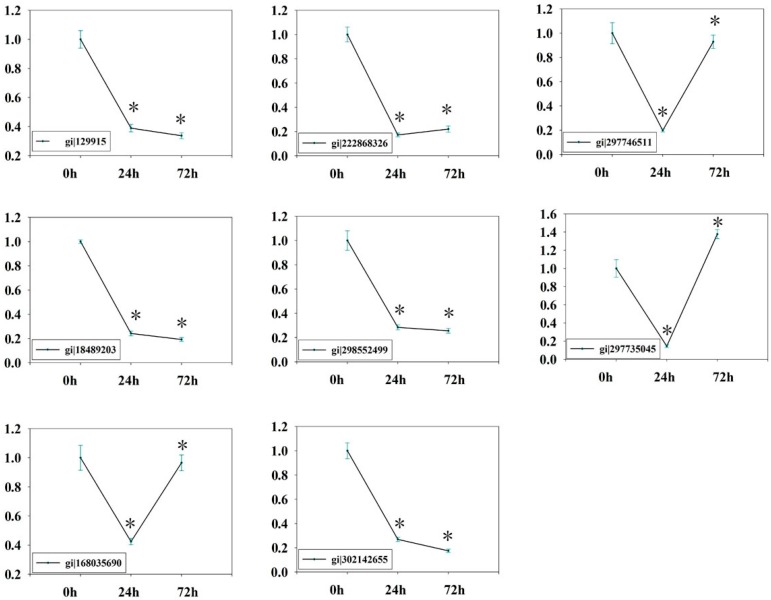
qPCR data for the mRNA expression levels of genes for drought-responsive proteins mapped in glycolytic/gluconeogenetic pathway in ramie leaves. The values represent relative mRNA levels against control groups (0 h samples), values of which were all set to 1 unit. Statistically significant differences in gene expression are indicated with asterisks: * *p* < 0.05.

**Figure 10:**
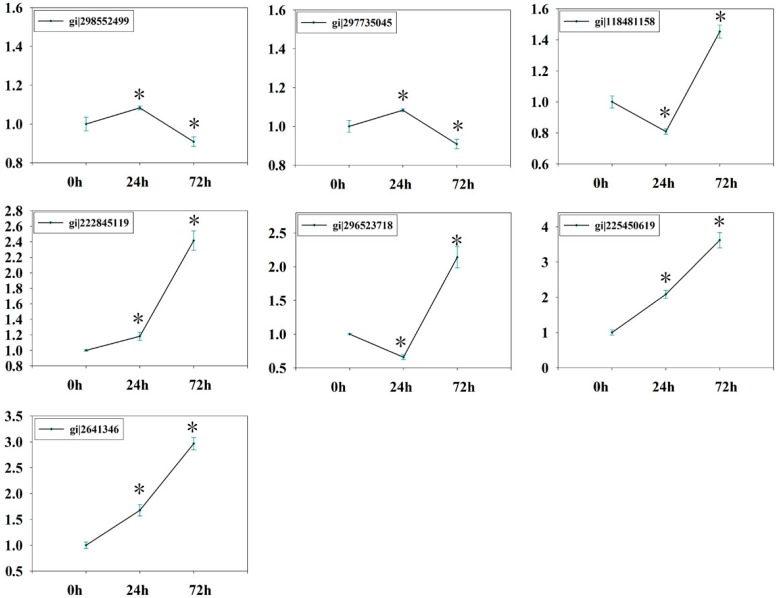
qPCR data for the mRNA expression levels of genes for drought-responsive proteins mapped in glycolytic/gluconeogenetic pathway in roots of ramie. The values represent relative mRNA levels against control groups (0 h samples), values of which were all set to 1 unit. Statistically significant differences in gene expression are indicated with asterisks: * *p* < 0.05.

To obtain information complementary to the iTRAQ data and KOBAS results, we also examined expression levels of genes involved in glycolysis/gluconeogenesis. RNA of the leaves and roots was extracted after 0 h, 24 h, and 72 h of PEG6000 treatments and subjected to qPCR analysis. qPCR data values for Figure 9 and Figure 10 are shown in Table S7, using *GAPDH* as an internal control. The expression patterns of the twelve genes (gi|302774424, gi|82941449, gi|298541583, gi|129915, gi|222868326, gi|297746511, gi|118489203, gi|298552499, gi|297735045, gi|222845119, gi|168035690, and gi|302142655) were summarized in Figure 8. The qPCR results show that, upon drought stress, most genes encoding key enzymes related to glycolysis/gluconeogenesis were significantly downregulated in leaves of ramie (Figure 9), in agreement with the iTRAQ data. However, gi|82941449 and gi|297735045 were downregulated after 24 h of treatment but upregulated after 72 h. These data indicate that the transcript and protein levels of differentially expressed genes are not always consistent. The expression patterns of the seven genes in roots (gi|298552499, gi|297735045, gi|118481158, gi|222845119, gi|296523718, gi|225450619, and gi|2641346) are summarized in Figure 10. Upon drought stress, most genes encoding key enzymes related to glycolysis/gluconeogenesis were significantly upregulated in roots (Figure 10), in line with the iTRAQ data. Such remarkable activation of glycolytic/gluconeogenetic pathway suggests a strong promotion of biosynthesis of available energy [34].

## 3. Materials and Methods

### 3.1. Plant Materials and Stress Treatments

“Huazhu No. 5” is an elite ramie variety [53] with characteristics of high yield, good fiber quality, and high drought resistance levels. Two weeks after planting, the “Huazhu No. 5” plantlets were propagated from stem cuttings, which were transplanted into a half-strength Hoagland's solution for 20 days. The seedling stage was considered to last until the plants reached about 10 cm in height (Figure 1a). Stem cuttings were prepared for ramie plantlets. Plantlets were used as sources of the leaves and roots. They were cultured under cool white fluorescent light in 16/8 h (light/dark) with a relative humidity of 50%–70% and temperatures about 25 ± 2 °C in the daytime and 20 ± 2 °C at night.

Leaf and root samples of the same size were harvested and photographed at 0 (L1 and R1; Figure 1b), 24 (L2 and R2; Figure 1c), and 72 (L3 and R3; Figure 1d) hours after the plantlets had been treated with 15% (*w*/*v*) PEG6000 to induce drought stress with three replicates. Half-strength Hoagland's solution was replaced each day with freshly prepared solutions. Leaves and roots were collected and frozen in liquid nitrogen and stored at −80 °C prior to analysis.

### 3.2. Protein Extraction

Proteins from two biological replicates in each treatment were extracted and prepared as previously described with some modifications [29]. Dry protein powder was treated with a 0.5-mL lysis buffer (8 M of urea, 4% CHAPS, 40 mM of Tris-HCL, 5 mM of EDTA (Sigma, St. Louis, MO, USA), 1 mM of PMSF (Sigma, St. Louis, MO, USA), and 10 mM of Dithiothreitol (DTT) (Sigma, St. Louis, MO, USA), pH 8.0). The samples were sonicated thrice for 5 min on ice using a high intensity ultrasonic processor. The remaining debris was removed by centrifugation at 30,000× *g* at 4 °C for 15 min. The supernatant was transferred to a new tube, reduced with 10 mM of DTT for 1 h, and alkylated with 55 m of Miodoacetamide for 45 min at room temperature in darkness. Then, 4 volumes of prechilled acetone were added to the protein, which was precipitated for 30 min at −20 °C. After centrifugation, the pellet was then dissolved in 0.5 M of TEAB (Sigma, St. Louis, MO, USA) and sonicated for 5 min. The centrifugation step was repeated, and the supernatant was collected. Protein content was determined with a 2-D Quant kit (GE Healthcare, Piscataway, NJ, USA) according to the manufacturer’s instructions.

### 3.3. Digestion and iTRAQ Labeling 

Approximately 100 μg of protein for each sample was digested with trypsin (Promega, Madison, WI, USA) overnight at 37 °C in a 1:20 trypsin-to-protein mass ratio. After trypsin digestion, peptides were dried by vacuum centrifugation, reconstituted in 0.5 M of TEAB, and processed with an 8-plex iTRAQ kit (Applied Biosystems, Foster City, CA, USA). Briefly, one unit of iTRAQ reagent (defined as the amount of reagent required to label 100 μg of peptides) was thawed and reconstituted in 70 μL of isopropanol. Peptides from treatment and control subgroups were labeled with different iTRAQ tags by incubation at room temperature for 2 h. The iTRAQ-labeled peptide mixtures were then pooled, dried by vacuum centrifugation (Speed-Vac, Savant) and fractionated by strong cationic exchange (SCX) chromatography (Phenomenex, Guangzhou, China).

### 3.4. Fractionation by Strong Cationic Exchange (SCX) Chromatography

For SCX chromatography using a Shimadzu LC-20AB HPLC Pump system (Kyoto, Japan), the iTRAQ-labeled peptide mixture was reconstituted with 4 mL of buffer A (25 mM of NaH_2_PO_4_ in 25% ACN, pH 3.0) and loaded onto a 4.6 × 250-mm Ultremex SCX column containing 5-μm particles (Phenomenex, Torrance, CA, USA). The peptides were eluted at a flow rate of 1 mL/min with a gradient of buffer A for 10 min, 5%–35% buffer B (25 mM of NaH_2_PO_4_, 1 M of KCl in 25% ACN, pH 3.0) for 11 min, and 35%–80% buffer B for 1 min. The system was then maintained in 80% buffer B for 3 min before equilibrating with buffer A for 10 min prior to the next injection. Elution was monitored by measuring absorbance at 214 nm, and fractions were collected every 1 min. The eluted peptides were pooled as 10 fractions, desalted with a Strata X C18 column (Phenomenex), and vacuum-dried.

### 3.5. Liquid Chromatography-Electrospray Ionization-Tandem Mass Spectrometry (LC-ESI-MS/MS) Analysis 

Each fraction was resuspended in 10 μL of buffer A (5% ACN, 0.1% FA) and centrifuged at 20,000 *g* for 10 min. In each fraction, the final concentration of peptide was about 0.5 µg/uL on average using a NanoDrop (Thermo Fisher Scientific, Wilmington, DE, USA) in conjunction with the Scopes method [54]. Supernatant (8 μL) was loaded on a Dionex Ultimate 3000 UPLC system by the autosampler onto a C18 trap column at 8 μL/min for 4 min, and the peptides were eluted onto an analytical C18 column (inner diameter 75 μm) packed in-house. The 40-min gradient was run at 300 nL/min starting from 2% to 35% B (95% ACN, 0.1% FA), followed by a 5-min linear gradient to 80%, maintenance at 80% B for 4 min, and a return to 5% in 1 min.

The peptides were subjected to nanoelectrospray ionization followed by tandem mass spectrometry (MS/MS) in a nano-ESI LTQ-Velos Pro Orbitrap-Elite mass spectrometer (ThermoFisher Scientific, San Jose, CA, USA) coupled online to the ultra performance liquid chromatography (UPLC). Intact peptides were detected in the Orbitrap at a resolution of 60,000. MS scans ranged from 350 to 2000 *m*/*z*. MS/MS was performed using a high energy collision dissociation (HCD) operating mode with a normalized collision energy setting of 45%. MS/MS spectra of up to 15 of the most intense ions were acquired. Isolation width was set as 2 *m*/*z* and dynamic exclusion was set as 30 s. The electrospray voltage applied was 1.8 kV. The mass spectrometry proteomics data have been deposited to the ProteomeXchange Consortium [55] via the PRIDE partner repository with the dataset identifier PXD001940 and 10.6019/PXD001940.

### 3.6. Association Analysis of Proteomics and Transcriptomics

In the ramie transcriptome sequencing, the de novo assembly often carried out under the circumstances, neither genomic information nor directly linked transcriptome information were available. In this study, we used previously published Illumina Paired-End sequencing project data, because the drought stress treatment on the material is the same as the material treatment of ramie transcriptome analysis we published previously [25]. Cluster analysis was used to identify groups of similarly differentially expressed proteins and transcripts at three drought stress stages, and the results output through the software Java Treeview in graphic form. Cluster analysis of association expression in differentially expressed proteins with corresponding transcript levels data was analyzed with Cluster 3.0.

### 3.7. Quantitative Realtime PCR (qRT-PCR)

Each sample comprised material from three plants that were mixed. Leaf and roots were collected and frozen in liquid nitrogen and stored at −80 °C prior to analysis for subsequent RNA extraction. Total RNA was separately isolated from the six samples (L1, L2, L3, R1, R2, and R3) with the RNAprep Pure Plant Kit (Tiangen Biotech, Beijing, China), following the manufacturer’s instructions. RNA quality was confirmed by gel electrophoresis and with the NanoDrop 2000 spectrophotometer (Thermo Fisher Scientific, Wilmington, DE, USA). The ramie *GAPDH* gene was selected as an internal control in each reaction. The primers used in this study were listed in Table 1. PCR amplification was performed as previously described [25]. The reactions were performed in triplicate, and the results were averaged.

### 3.8. Data Analysis

The raw mass data were converted to anmgf.file with Proteome Discover 1.3 (Thermo Fisher Scientific) with in-house MASCOT software 2.3.02 (Matrix Science, London, UK). In the database search, full tryptic specificity was required with tolerance set at one missed cleavage. The FDR (false positive rate) was cutoff with 1%. In this study, hierarchical cluster analyses were conducted according to a previous report [28]. Carbamido methylation of cysteine was set as a fixed modification. Gln->pyro-Glu of the N terminus, deamination of the N terminus, and oxidation of methionine were set as variable modifications. The initial precursor mass tolerance was set to 15 ppm, and the fragment ion level was set to 0.02 Da. iTRAQ 8-plex was set as quantitation. In this study, the database was obtained from transcription sequence [25]. The transcription sequence data have been deposited at the NCBI in the Short Read Archive database under accession SRP041143. After stringent quality checking and data cleaning, approximately 33,976,322,460 bp (30 G) of high-quality data (94.02% of the raw data) were generated under the Q20 standard (Q30 = 87.19%). Assembly of the high-quality sequencing reads yielded 138,381 unigenes, with an average length of 730.6 bp and a range from 201 to 20,860 bp. The N50 scaffold size was 972 bp. The unigene sequences were compared to the non-redundant (nr) protein database with a cutoff E-value of 1 × 10^−5^. As a result, 47,565 unigenes (34%) were annotated. The cutoff value for downregulated proteins was 0.67-fold and for the upregulated proteins was 1.5-fold [56]. The *p* value threshold was <0.05. The differentially expressed proteins were used for GO terms/KEGG pathway enrichment analyses using the hyper geometric test to measure significantly enriched terms:

$$P=1-\sum_{i=0}^{m-1}\frac{\left(\begin{array}{l} M\\ i \end{array}\right)\left(\begin{array}{l} N-M\\ n-i \end{array}\right)}{\left(\begin{array}{l} N\\ n \end{array}\right)}$$

where *N* is the number of proteins with GO/KEGG annotations, and n represents the number of differentially expressed proteins in *N*. The variables, *M* and *m*, represent the total number of proteins and the number of differentially expressed proteins, respectively, in each GO/KEGG term. The threshold for significant enrichment of protein sets was corrected to a *p* value of ≤0.05. Every experiment was carried out with three biological replicates, except iTRAQ experiments, carried out with two biological replicates.

## 4. Conclusions

The iTRAQ-based comparative proteome analysis and qPCR data presented here will help in further understanding the responses of ramie to drought stress and improving the drought tolerance of this fiber crop. KOBAS analysis indicated that glycolysis/gluconeogenesis was the main metabolic pathway upregulated in ramie roots in response to drought stress. The activation of glycolysis/gluconeogenesis in ramie roots appeared to be a rapid and effective way to balance the levels of available energy to prevent intracellular energy shortages. In conclusion, this study provides a valuable source for proteomic studies in ramie plants, especially in those under drought stress. Our work will assist in breeding drought-resistant ramie varieties.

## Figures and Tables

**Table 1:** Numbers of differently expressed proteins during drought stress. The “upregulated” row indicates the number of upregulated proteins in posterior samples compared with anterior samples. The “downregulated” row indicates the number of downregulated proteins in posterior samples compared with anterior samples.

Samples	Upregulated	Downregulated
L1–L2	10	108
L2–L3	0	216
L1–L3	20	413
R1–R2	122	2
R2–R3	20	7
R1–R3	211	29

## Supplementary Materials

The mass spectrometry proteomics data have been deposited to the Proteome Xchange Consortium [55] via the PRIDE partner repository with the dataset identifier PXD001940 and 10.6019/PXD001940. Supplementary materials can be found at www.mdpi.com/1422-0067/17/10/1607/s1.

## Abbreviations

iTRAQ	isobaric tag for relative and absolute quantification
SCX	strong cation exchange
HPLC	high pressure liquid chromatography
KEGG	Kyoto Encyclopedia of Genes and Genomes
KOBAS	KEGG Orthology-Based Annotation System
PEG	polyethylene glycol
RWC	relative water content
POD	peroxidaseactivity
qRT-PCR	quantitative realtime PCR

## References

[1] Mohammadi P.P., Moieni A., Komatsu S. (2012). Comparative proteome analysis of drought-sensitive and drought-tolerant rapeseed roots and their hybrid F1 line under drought stress. Amino Acids.

[2] Hu H., Dai M., Yao J., Xiao B., Li X., Zhang Q., Xiong L. (2006). Overexpressing a NAM, ATAF, and CUC (NAC) transcription factor enhances drought resistance and salt tolerance in rice. Proc. Natl. Acad. Sci. USA.

[3] Alam I., Lee D.G., Kim K.H., Park C.H., Sharmin S.A., Lee H., Oh K.W., Yun B.W., Lee B.H. (2010). Proteome analysis of soybean roots under waterlogging stress at an early vegetative stage. J. Biosci..

[4] Zhang Q. (2007). Strategies for developing green super rice. Proc. Natl. Acad. Sci. USA.

[5] Zhu J.K. (2002). Salt and drought stress signal transduction in plants. Annu. Rev. Plant Biol..

[6] Shinozaki K., Yamaguchi-Shinozaki K., Seki M. (2003). Regulatory network of gene expression in the drought and cold stress responses. Curr. Opin. Plant Biol..

[7] Seki M., Kamei A., Yamaguchi-Shinozaki K., Shinozaki K. (2003). Molecular responses to drought, salinity and frost: Common and different paths for plant protection. Curr. Opin. Biotechnol..

[8] Plomion C., Lalanne C., Claverol S., Meddour H., Kohler A., Bogeat-Triboulot M.B., Barre A., Le Provost G., Dumazet H., Jacob D. (2006). Mapping the proteome of poplar and application to the discovery of drought-stress responsive proteins. Proteomics.

[9] Aranjuelo I., Molero G., Erice G., Avice J.C., Nogues S. (2011). Plant physiology and proteomics reveals the leaf response to drought in alfalfa (*Medicago sativa* L.). J. Exp. Bot..

[10] Ashoub A., Beckhaus T., Berberich T., Karas M., Bruggemann W. (2013). Comparative analysis of barley leaf proteome as affected by drought stress. Planta.

[11] Matsui A., Ishida J., Morosawa T., Mochizuki Y., Kaminuma E., Endo T.A., Okamoto M., Nambara E., Nakajima M., Kawashima M. (2008). *Arabidopsis* transcriptome analysis under drought, cold, high-salinity and aba treatment conditions using a tiling array. Plant Cell Physiol..

[12] Zheng J., Fu J., Gou M., Huai J., Liu Y., Jian M., Huang Q., Guo X., Dong Z., Wang H. (2010). Genome-wide transcriptome analysis of two maize inbred lines under drought stress. Plant Mol. Biol..

[13] Cal A.J., Liu D., Mauleon R., Hsing Y.I., Serraj R. (2013). Transcriptome profiling of leaf elongation zone under drought in contrasting rice cultivars. PLoS ONE.

[14] Le D.T., Nishiyama R., Watanabe Y., Tanaka M., Seki M., Ham le H., Yamaguchi-Shinozaki K., Shinozaki K., Tran L.S. (2012). Differential gene expression in soybean leaf tissues at late developmental stages under drought stress revealed by genome-wide transcriptome analysis. PLoS ONE.

[15] Liu T., Zhu S., Tang Q., Yu Y., Tang S. (2013). Identification of drought stress-responsive transcription factors in ramie (*Boehmeria nivea* L. Gaud.). BMC Plant Biol..

[16] Budak H., Akpinar B.A., Unver T., Turktas M. (2013). Proteome changes in wild and modern wheat leaves upon drought stress by two-dimensional electrophoresis and nanolc-esi-ms/ms. Plant Mol. Biol..

[17] Liu F., Liu Q., Liang X., Huang H., Zhang S. (2005). Morphological, anatomical, and physiological assessment of ramie (*Boehmeria nivea* (L.) Gaud.) tolerance to soil drought. Genet. Resour. Crop Evol..

[18] Bonhomme L., Monclus R., Vincent D., Carpin S., Lomenech A.M., Plomion C., Brignolas F., Morabito D. (2009). Leaf proteome analysis of eight populus xeuramericana genotypes: Genetic variation in drought response and in water-use efficiency involves photosynthesis-related proteins. Proteomics.

[19] Ali G.M., Komatsu S. (2006). Proteomic analysis of rice leaf sheath during drought stress. J. Proteome Res..

[20] Zang X., Komatsu S. (2007). A proteomics approach for identifying osmotic-stress-related proteins in rice. Phytochemistry.

[21] Nouri M.Z., Komatsu S. (2010). Comparative analysis of soybean plasma membrane proteins under osmotic stress using gel-based and LC MS/MS-based proteomics approaches. Proteomics.

[22] Yang L.T., Qi Y.P., Lu Y.B., Guo P., Sang W., Feng H., Zhang H.X., Chen L.S. (2013). iTRAQ protein profile analysis of citrus sinensis roots in response to long-term boron-deficiency. J. Proteom..

[23] Zieske L.R. (2006). A perspective on the use of itraq reagent technology for protein complex and profiling studies. J. Exp. Bot..

[24] Verslues P.E., Agarwal M., Katiyar-Agarwal S., Zhu J., Zhu J.K. (2006). Methods and concepts in quantifying resistance to drought, salt and freezing, abiotic stresses that affect plant water status. Plant J..

[25] An X., Chen J., Zhang J., Liao Y., Dai L., Wang B., Liu L., Peng D. (2015). Transcriptome profiling and identification of transcription factors in ramie (*Boehmeria nivea* L. Gaud) in response to peg treatment, using illumina paired-end sequencing technology. Int. J. Mol. Sci..

[26] Ashraf M. (2009). Biotechnological approach of improving plant salt tolerance using antioxidants as markers. Biotechnol. Adv..

[27] Chen S., Cui X., Chen Y., Gu C., Miao H., Gao H., Chen F., Liu Z., Guan Z., Fang W. (2011). Cgdreba transgenic chrysanthemum confers drought and salinity tolerance. Environ. Exp. Bot..

[28] Zi J., Zhang J., Wang Q., Zhou B., Zhong J., Zhang C., Qiu X., Wen B., Zhang S., Fu X. (2013). Stress responsive proteins are actively regulated during rice (*Oryza sativa*) embryogenesis as indicated by quantitative proteomics analysis. PLoS ONE.

[29] Deng G., Liu L.J., Zhong X.Y., Lao C.Y., Wang H.Y., Wang B., Zhu C., Shah F., Peng D.X. (2014). Comparative proteome analysis of the response of ramie under N, P and K deficiency. Planta.

[30] Jiang Y., Yang B., Harris N.S., Deyholos M.K. (2007). Comparative proteomic analysis of nacl stress-responsive proteins in *Arabidopsis* roots. J. Exp. Bot..

[31] Ge P., Ma C., Wang S., Gao L., Li X., Guo G., Ma W., Yan Y. (2012). Comparative proteomic analysis of grain development in two spring wheat varieties under drought stress. Anal. Bioanal. Chem..

[32] Wang N., Zhao J., He X., Sun H., Zhang G., Wu F. (2015). Comparative proteomic analysis of drought tolerance in the two contrasting tibetan wild genotypes and cultivated genotype. BMC Genom..

[33] Peng Z., Wang M., Li F., Lv H., Li C., Xia G. (2009). A proteomic study of the response to salinity and drought stress in an introgression strain of bread wheat. Mol. Cell. Proteom..

[34] Wang Z.Q., Xu X.Y., Gong Q.Q., Xie C., Fan W., Yang J.L., Lin Q.S., Zheng S.J. (2014). Root proteome of rice studied by iTRAQ provides integrated insight into aluminum stress tolerance mechanisms in plants. J. Proteom..

[35] Nogues S., Baker N.R. (2000). Effects of drought on photosynthesis in mediterranean plants grown under enhanced uv-b radiation. J. Exp. Bot..

[36] Ramachandra Reddy A., Chaitanya K.V., Vivekanandan M. (2004). Drought-induced responses of photosynthesis and antioxidant metabolism in higher plants. J. Plant Physiol..

[37] Maksup S., Roytrakul S., Supaibulwatana K. (2012). Physiological and comparative proteomic analyses of thai jasmine rice and two check cultivars in response to drought stress. J. Plant Int..

[38] Vassileva V., Demirevska K., Simova-Stoilova L., Petrova T., Tsenov N., Feller U. (2012). Long-term field drought affects leaf protein pattern and chloroplast ultrastructure of winter wheat in a cultivar-specific manner. J. Agron. Crop Sci..

[39] Caruso G., Cavaliere C., Foglia P., Gubbiotti R., Samperi R., Laganà A. (2009). Analysis of drought responsive proteins in wheat (*Triticum durum*) by 2D-page and maldi-tof mass spectrometry. Plant Sci..

[40] Guo X.L., Bai L.R., Su C.Q., Shi L.R., Wang D.W. (2013). Molecular cloning and expression of drought-induced protein 3 (DIP3) encoding a class III chitinase in upland rice. Genet. Mol. Res..

[41] Shinozaki K., Yamaguchi-Shinozaki K. (2000). Molecular responses to dehydration and low temperature: Differences and cross-talk between two stress signaling pathways. Curr. Opin. Plant Biol..

[42] Finkelstein R.R., Gampala S.S., Rock C.D. (2002). Abscisic acid signaling in seeds and seedlings. Plant Cell.

[43] Xiong L., Schumaker K.S., Zhu J.K. (2002). Cell signaling during cold, drought, and salt stress. Plant Cell.

[44] Yao Y., Ni Z., Du J., Wang X., Wu H., Sun Q. (2006). Isolation and characterization of 15 genes encoding ribosomal proteins in wheat (*Triticum aestivum* L.). Plant Sci..

[45] Swindell W.R., Huebner M., Weber A.P. (2007). Transcriptional profiling of *Arabidopsis* heat shock proteins and transcription factors reveals extensive overlap between heat and non-heat stress response pathways. BMC Genom..

[46] Xie C., Mao X., Huang J., Ding Y., Wu J., Dong S., Kong L., Gao G., Li C.Y., Wei L. (2011). Kobas 2.0: A web server for annotation and identification of enriched pathways and diseases. Nucleic Acids Res..

[47] Baena-Gonzalez E., Rolland F., Thevelein J.M., Sheen J. (2007). A central integrator of transcription networks in plant stress and energy signalling. Nature.

[48] Smith A.M., Stitt M. (2007). Coordination of carbon supply and plant growth. Plant Cell Environ..

[49] Baena-Gonzalez E., Sheen J. (2008). Convergent energy and stress signaling. Trends Plant Sci..

[50] Hamilton C.A., Good A.G., Taylor G.J. (2001). Induction of vacuolar atpase and mitochondrial ATP synthase by aluminum in an aluminum-resistant cultivar of wheat. Plant Physiol..

[51] Shi D.Y., Xie F.Z., Zhai C., Stern J.S., Liu Y., Liu S.L. (2009). The role of cellular oxidative stress in regulating glycolysis energy metabolism in hepatoma cells. Mol. Cancer.

[52] Ouyang S.Q., Liu Y.F., Liu P., Lei G., He S.J., Ma B., Zhang W.K., Zhang J.S., Chen S.Y. (2010). Receptor-like kinase ossik1 improves drought and salt stress tolerance in rice (*Oryza sativa*) plants. Plant J..

[53] An X., Wang B., Liu L., Jiang H., Chen J., Ye S., Chen L., Guo P., Huang X., Peng D. (2014). Agrobacterium-mediated genetic transformation and regeneration of transgenic plants using leaf midribs as explants in ramie (*Boehmeria nivea* (L.) Gaud). Mol. Biol. Rep..

[54] Villeneuve L.M., Stauch K.L., Fox H.S. (2014). Proteomic analysis of the mitochondria from embryonic and postnatal rat brains reveals response to developmental changes in energy demands. J. Proteom..

[55] Vizcaino J.A., Deutsch E.W., Wang R., Csordas A., Reisinger F., Rios D., Dianes J.A., Sun Z., Farrah T., Bandeira N. (2014). Proteomexchange provides globally coordinated proteomics data submission and dissemination. Nat. Biotechnol..

[56] Fan N.J., Gao C.F., Wang C.S., Zhao G., Lv J.J., Wang X.L., Chu G.H., Yin J., Li D.H., Chen X. (2012). Identification of the up-regulation of TP-α, collagen α-1(VI) chain, and S100A9 in esophageal squamous cell carcinoma by a proteomic method. J. Proteom..

