# Supplementary material for: Isobaric Tags for Relative and Absolute Quantitation (iTRAQ)-Based Comparative Proteome Analysis of the Response of Ramie under Drought Stress
Source: Int J Mol Sci. 2016 Sep 27;17(10):1607. doi: 10.3390/ijms17101607 (PMC5085640; doi:10.3390/ijms17101607)
Supplement: Supplementary file 1 [file ijms-17-01607-s001.zip › ijms-134229-Supplementary Materials/ijms-134229-supplementary.pdf]

Supplementary Materials: Isobaric Tags for Relative and Absolute Quantitation (iTRAQ)-Based Comparative Proteome Analysis of the Response of Ramie under Drought Stress

Xia An, Jingyu Zhang, Lunjin Dai, Gang Deng, Yiwen Liao, Lijun Liu, Bo Wang and Dingxiang Peng

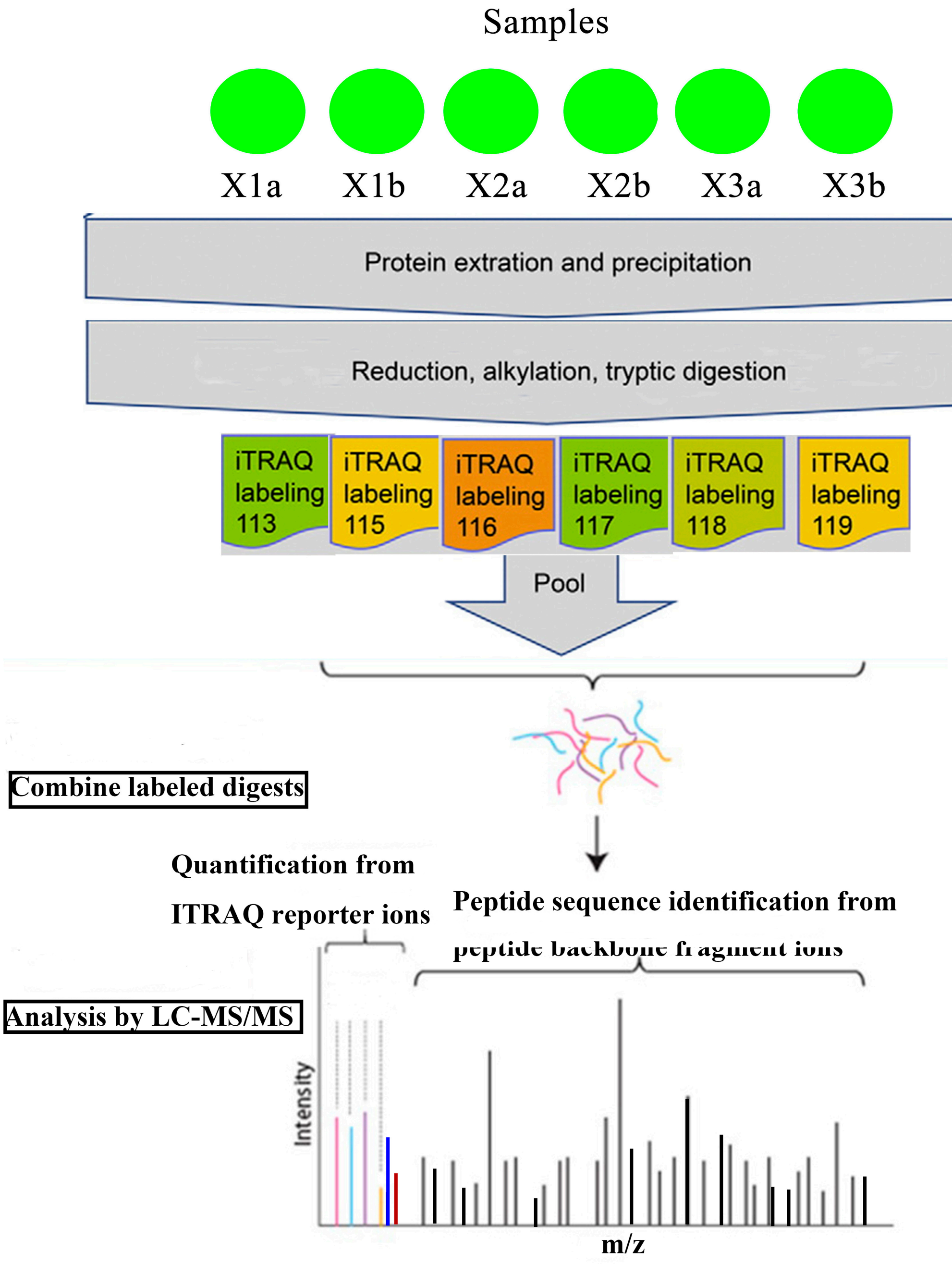

Figure S1. A flow chart of the iTRAQ experiment for quantitative proteome analysis [1]. X means leaves or roots of ramie. X1 means L1 or R1; X2 means L2 or R2; X3 means L3 or R3; a and b represent two repeats, respectively.

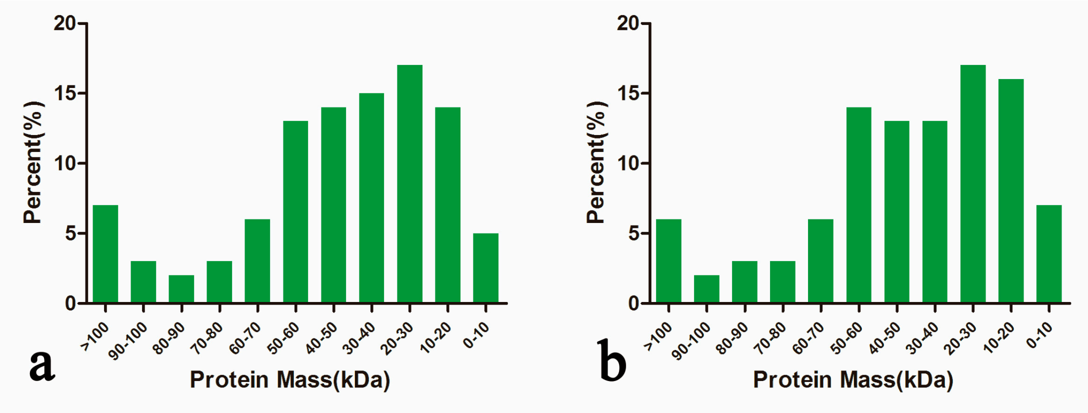

Figure S2. Protein mass distribution in ramie **a** in leaves; **b** in roots.

Table S1. Details for 44 important functional groups.

Table S2. All leaf proteins and root proteins were classified into 22 COG subcategories and 21 subcategories, respectively.

Table S3. Details for hierarchical display of data from differentially expressed protein of leaves under drought stress.

Table S4. Details for hierarchical display of data from differentially expressed protein of roots under drought stress.

Table S5. Differentially expressed proteins of leaves and roots under drought stress.

Table S6. Alternative bioenergetic pathways in stressed organisms.

Table S7. qPCR data values for Figure 9 and 10.

References

1 Wang, Z.Q.; Xu, X.Y.; Gong, Q.Q.; Xie, C.; Fan, W.; Yang, J.L.; Lin, Q.S.; Zheng, S.J. Root proteome of rice studied by iTRAQ provides integrated insight into aluminum stress tolerance mechanisms in plants. *J. Proteom.* **2014**, *98*, 189–205.
